# Clinical and Radiographic Success of Pulpotomy in Permanent Teeth With Irreversible Pulpitis: A Systematic Review

**DOI:** 10.7759/cureus.116052

**Published:** 2026-09-10

**Authors:** Omar A Almohsen

**Affiliations:** 1 Pediatric Dentistry, Private Practice, Qassim, SAU

**Keywords:** biodentine, clinical success, irreversible pulpitis, mta, permanent teeth, pulpotomy, radiographic success, vital pulp therapy

## Abstract

Irreversible pulpitis in permanent teeth has traditionally been managed by root canal treatment. However, currently, vital pulp therapy, especially full and partial pulpotomy, is gaining popularity as a conservative technique to maintain pulp vitality and preserve tooth structure.

This systematic review was carried out following the guidelines of the PRISMA 2020. A literature search was conducted using electronic databases PubMed, Cochrane library, and Google Scholar for studies published from 2010 to 2026. Following screening and eligibility assessment according to predefined inclusion criteria, 16 studies were included for qualitative synthesis.

A total of 375 records were identified. Following removal of duplicates and screening, 16 studies were eligible and included in the review. Most included studies were randomized controlled trials, while a few were prospective or retrospective cohort studies. Overall, the included studies consistently reported favorable clinical and radiographic outcomes following pulpotomy in permanent teeth with irreversible pulpitis, particularly when calcium silicate-based materials such as mineral trioxide aggregate (MTA), Biodentine (Septodont, Saint-Maur-des-Fossés, France), calcium-enriched mixture (CEM), TotalFill (FKG Dentaire, La Chaux-de-Fonds, Switzerland), and other bioceramics were used.

Pulpotomy appears to be an effective and predictable conservative treatment option for permanent teeth with irreversible pulpitis, demonstrating favorable clinical and radiographic outcomes across both short- and long-term follow-up periods. However, additional high-quality multicenter studies with extended follow-up are required to further strengthen treatment recommendations.

## Introduction and background

Irreversible pulpitis is one of the most common pulpal abnormalities seen in permanent teeth and is often seen together with deep caries, bacterial penetration and pulpal inflammation. Clinically, it is characterized by spontaneous pain, long duration of thermal sensitivity and symptoms of chronic pulpal inflammation. Irreversible pulpitis has long been regarded as a sign of pulp destruction, and hence a sign for complete pulpal removal and root canal treatment (RCT) to preserve the teeth with impaired pulpal viability [1,2].

While conventional RCT offers good success rates, it can be more complex, chairside time can be longer, more expensive, and significant tooth structure must be removed. In addition, root canal treatment may not always be easily accessible in all health care environments and may be difficult in young people and medically compromised patients [3]. These limitations have increased interest in minimally invasive and biologically based treatment approaches aimed at preserving pulp vitality whenever possible.

The traditional understanding of irreversible pulpitis is that the inflamed pulp tissue is incapable of healing. This premise has been challenged by advances in the understanding of the biology of pulp. Histological findings indicate that inflammation is likely to be confined to the coronal pulp area, and the radicular pulp may be healthy or have a regeneration potential. This changing perspective has led to a renewed interest in vital pulp therapy (VPT) as a conservative option to traditional endodontics [4,5].

Pulpotomy is a specific type of VPT, where the inflamed coronal pulp tissue is removed, but the remaining radicular pulp is maintained to be vital and functional. The extent of the removal of the tissues determines if the pulpotomy is performed as partial pulpotomy, full pulpotomy, deep pulpotomy, or partial radicular pulpotomy. Currently, bioactive materials like mineral trioxide aggregate (MTA), Biodentine (Septodont, Saint-Maur-des-Fossés, France), calcium-enriched mixture (CEM) cement, calcium silicate-based biomaterials and other bioceramic agents are widely used, as they have demonstrated their biocompatibility, sealing ability, antibacterial properties, dentin bridge formation and pulp healing capabilities [6,7].

In recent years, the clinical guidelines and position statements of endodontic organizations have begun to accept the importance of vital pulp therapy in permanent teeth with pulpal exposure. Vital pulp therapy should be considered in selected permanent teeth with symptoms and signs of irreversible pulpitis when adequate haemostasis is provided, aseptic technique is maintained, biomaterials are used correctly and coronal seal is provided [8,9]. This paradigm shift is due to the focus on minimal intervention dentistry and maintenance of pulp vitality, rather than pulp removal.

Although the acceptance of pulpotomy has grown, there remains significant heterogeneity in terms of pulpotomy technique, materials, patient selection, diagnostic criteria, length of follow-up, and definitions of pulpotomy success. The lack of clinical and radiographic predictability of pulpotomy for irreversible pulpitis in permanent teeth has helped maintain the uncertainty about its definitive treatment status [10]. In addition, while there are numerous clinical studies reporting positive results, studies are found in varying study designs and intervention protocols.

A systematic review is warranted to summarize all the available evidence of the clinical and radiographic outcomes of pulpotomy in the diagnosis of irreversible pulpitis from permanent teeth. Thus, this systematic review aimed to analyze the clinical and radiographic outcomes of pulpotomy in permanent teeth with irreversible pulpitis and its possible use as a conservative treatment in comparison to conventional root canal therapy.

## Review

Methods

Study Design and Reporting Guidelines

This systematic review was conducted in accordance with the Preferred Reporting Items for Systematic Reviews and Meta-Analyses (PRISMA) 2020 guidelines [11]. The review protocol was formulated before selection of the studies, and the search strategy used was designed based on the Population, Intervention, Comparison, and Outcomes (PICO) framework (Table 1).

**Table 1:** PICO Framework of the Review

Component	Description
P (Population)	Patients with permanent teeth diagnosed with irreversible pulpitis (mature and/or immature permanent teeth)
I (Intervention)	Partial pulpotomy or full pulpotomy using materials such as Mineral Trioxide Aggregate (MTA), Biodentine, calcium silicate–based materials, calcium hydroxide, and bioceramic materials
C (Comparison)	Root canal treatment (RCT), other vital pulp therapies, different pulpotomy materials or techniques, or baseline status where applicable
O (Outcomes)	Clinical success (absence of pain, swelling, tenderness to percussion, sinus tract, retreatment) and radiographic success (absence of periapical radiolucency, periodontal ligament widening, root resorption, or pathological changes)

Eligibility Criteria

Studies were selected according to predefined inclusion and exclusion criteria.

Inclusion criteria:

1. Studies involving human participants with permanent teeth diagnosed with irreversible pulpitis. 2. Studies evaluating pulpotomy procedures (partial pulpotomy, full pulpotomy, deep pulpotomy, or partial radicular pulpotomy) as treatment for irreversible pulpitis, including different pulpotomy materials or comparisons with root canal treatment (RCT) or other vital pulp therapies where applicable. 3. Studies reporting clinical and/or radiographic outcomes, including pain relief, symptom resolution, pulp vitality preservation, and radiographic healing. 4. Randomized controlled trials, prospective studies, retrospective studies, and cohort studies. 5. Articles published between 2010 and 2026. 6. Articles published in the English language.

Exclusion criteria:

1. In vitro and animal studies. 2. Case reports, case series, narrative reviews, systematic reviews, meta-analyses, editorials, letters, conference abstracts, and study protocols. 3. Studies involving primary teeth or teeth without irreversible pulpitis. 4. Studies not related to pulpotomy in permanent teeth with irreversible pulpitis. 5. Studies lacking relevant clinical or radiographic outcome data. 6. Duplicate or overlapping publications. 7. Articles not published in English.

Information Sources and Search Strategy

The electronic search of PubMed, Cochrane Library (CENTRAL) and Google Scholar was performed for the period of January 2010 to January 2026. Using Boolean operators (AND/OR), search terms were created from combinations of keywords, including irreversible pulpitis, pulpotomy, vital pulp therapy, permanent teeth, clinical success, and radiographic success. Table 2 shows the detailed search strategy of each database.

**Table 2:** Search Strategy for Electronic Databases

Database	Search Strategy
PubMed	("irreversible pulpitis" OR "symptomatic irreversible pulpitis") AND (pulpotomy OR "partial pulpotomy" OR "full pulpotomy" OR "vital pulp therapy") AND ("permanent teeth" OR "permanent tooth" OR "mature permanent teeth" OR "immature permanent teeth") AND ("clinical success" OR "radiographic success" OR outcome OR prognosis OR survival). Filters applied: English language; publication years 2010–2026.
Cochrane Library (CENTRAL)	("irreversible pulpitis" OR "symptomatic irreversible pulpitis") AND (pulpotomy OR "partial pulpotomy" OR "full pulpotomy" OR "vital pulp therapy") AND ("permanent teeth" OR "permanent tooth" OR "mature permanent teeth" OR "immature permanent teeth") AND ("clinical success" OR "radiographic success" OR outcome OR prognosis OR survival). Publication period: 2010–2026; study type: trials (CENTRAL database).
Google Scholar	A supplementary search was conducted using keywords related to irreversible pulpitis, pulpotomy, permanent teeth, clinical success, and radiographic success. The first 200 results sorted by relevance were screened, and potentially eligible studies were assessed for inclusion.

A supplementary search was also conducted in Google Scholar by selecting the first 200 results sorted by relevance, and screening these for potentially eligible studies. Reference lists of selected studies were manually reviewed to identify additional relevant articles.

Search results were refined using publication year limits, English-language restriction, duplicate removal, and title/abstract relevance screening prior to full-text eligibility assessment. For Google Scholar, the first 200 relevance-ranked records were screened in accordance with the predefined search strategy.

Study Selection

All identified records were entered into EndNote 9 (Clarivate, London, UK) reference management software, and duplicate studies were removed. A total of 375 records were identified through database searching, including PubMed (n = 98), Cochrane Library (n = 77), and Google Scholar (n = 200). After removal of duplicate records (n = 58), 317 studies remained for title and abstract screening. Following screening, 47 full-text articles were assessed for eligibility, of which 31 studies were excluded for not meeting inclusion criteria, lack of relevant outcome data, duplicate publications, overlapping populations, and inability to extract data. Finally, 16 studies met the eligibility criteria and were included in this systematic review. The study selection process is illustrated in the PRISMA 2020 flow diagram.

Data Extraction

Data was extracted manually using a standardized data extraction form created in Microsoft Excel (Microsoft Corp., Redmond, WA, USA). Data extracted comprised study characteristics (author, year, country), study design, sample size, characteristics of the population, intervention and comparator (if applicable), pulpotomy material, follow-up duration, primary outcomes, major findings, and adverse effects reported. Extracted data were examined thoroughly to assure consistency, accuracy and completeness of data across all of the studies included.

Risk of Bias Evaluation

The methodological quality of the included studies was assessed manually. Randomized controlled trials were assessed using the Cochrane Risk of Bias tool (RoB 2), and observational studies (retrospective and prospective cohort studies) were assessed using the Newcastle-Ottawa Scale (NOS) [12]. Studies were assessed for the methodological areas that were relevant, and an overall judgement of risk of bias was subsequently made. Any uncertainties were resolved through detailed assessment of study methodology and reported findings.

Summary Measures

Summary measures included clinical success, radiographic success, postoperative pain reduction, pulp vitality preservation, hemostasis, and long-term treatment outcomes. Clinical success was defined as absence of pain, swelling, tenderness to percussion, sinus tract formation, retreatment, or symptoms, while radiographic success included absence or healing of periapical radiolucency, preservation of periodontal ligament (PDL) integrity, and absence of pathological root changes.

Data Synthesis

Although meta-analysis was initially considered, quantitative pooling was deemed inappropriate because of substantial clinical and methodological heterogeneity across studies, including differences in pulpotomy techniques (partial, full, deep, and radicular pulpotomy), biomaterials used (MTA, Biodentine, CEM, TotalFill and others), follow-up durations (6 months-5 years), outcome definitions, and study designs (RCTs and retrospective cohorts). Additionally, several studies did not report sufficiently homogeneous outcome measures or comparable statistical parameters required for pooled quantitative synthesis.

Results

Study Selection

A total of 375 records were identified through database searching, including PubMed (n = 98), Cochrane Library (n = 77), and Google Scholar (n = 200). Following removal of duplicate studies (n = 58), 317 studies were screened by title and abstract. Of these, 270 studies were excluded for not meeting the inclusion criteria.

A total of 47 full-text articles were reviewed for eligibility. After full-text assessment, 31 studies were excluded for reasons including non-eligibility, insufficient outcome data, duplicate publications, overlapping populations, and inability to extract relevant data. Finally, 16 studies met the eligibility criteria and were included in the systematic review (Figure 1).

**Figure 1:**
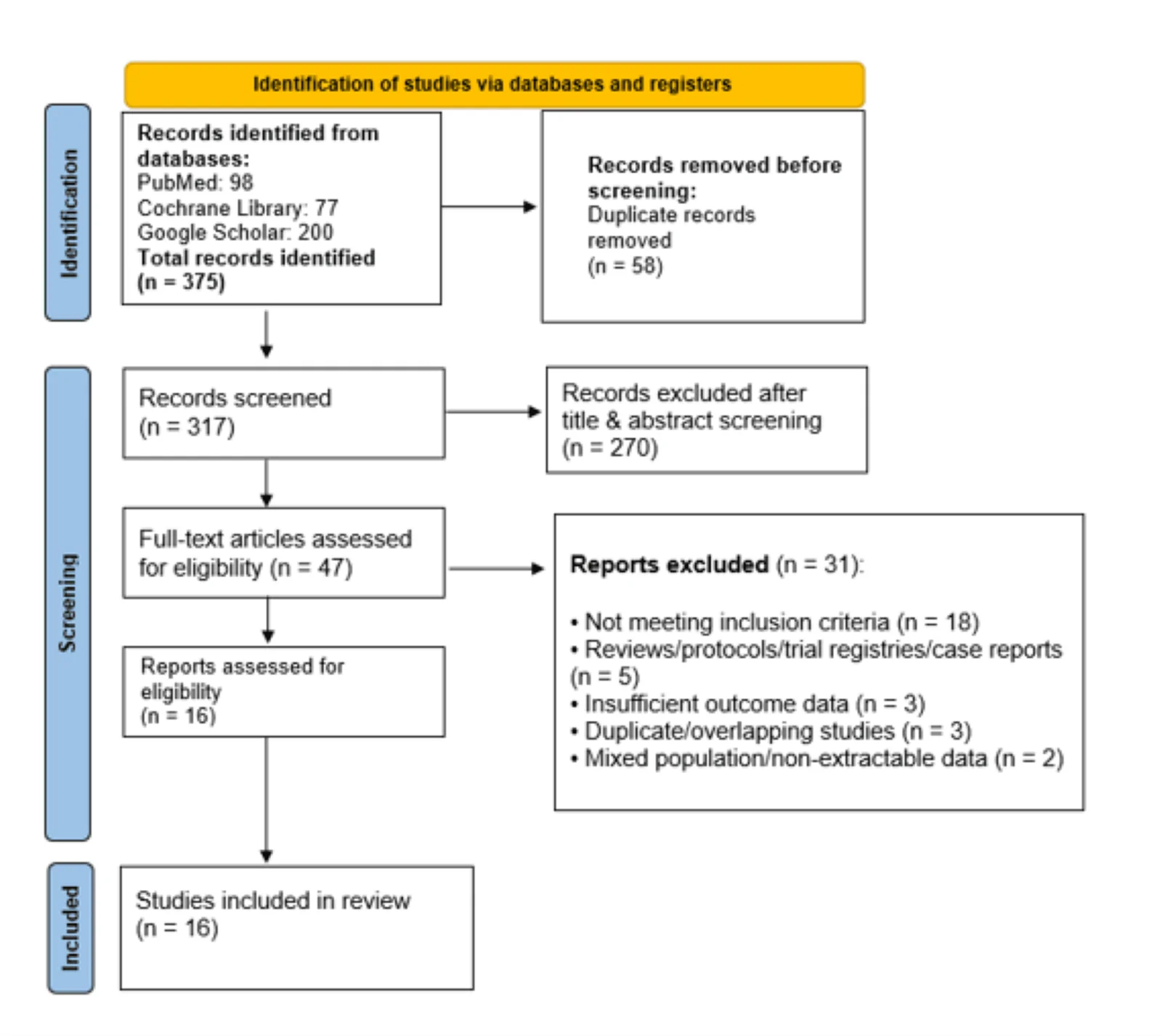
PRISMA 2020 flow diagram of study selection PRISMA: Preferred Reporting Items for Systematic Reviews and Meta-Analyses

Study Characteristics

Of the 16 included studies, 13 were randomized controlled trials, two were retrospective cohort studies, and one was a prospective cohort study. The studies have been carried out in various geographical areas such as India, Jordan, Iran, China, Greece, Pakistan, Senegal and Spain.

The number of participants in the studies ranged widely from 43 to 412. Among the included studies, MTA was evaluated in eight studies, Biodentine (Septodont, Saint-Maur-des-Fossés, France) in seven studies, calcium-enriched mixture (CEM) cement in two studies, TotalFill (FKG Dentaire, La Chaux-de-Fonds, Switzerland) in two studies, TheraCal PT (Bisco, Schamburg, IL, USA) in one study, EndoSequence Root Repair Material (ERRM) in one study, while other calcium silicate-based bioceramic materials were evaluated in selected studies. The majority of studies included mature permanent teeth diagnosed with symptomatic irreversible pulpitis (SIP), and a few studies included teeth with deep carious lesions, apical periodontitis or adult permanent teeth with irreversible pulpitis.

The pulpotomy materials used across the included studies included mineral trioxide aggregate (MTA), Biodentine, calcium-enriched mixture (CEM) cement, TotalFill, TheraCal PT, EndoSequence Root Repair Material (ERRM), calcium silicate-based materials, and other bioceramic materials, and the different intervention techniques evaluated were full pulpotomy, partial pulpotomy, deep pulpotomy, and partial radicular pulpotomy. Various studies have compared various pulpotomy techniques, biomaterials, or pulpotomy with root canal treatment, or evaluated pulpotomy as a single treatment. The follow-up duration across included studies ranged from 6 to 60 months, with studies reporting outcomes at 6, 12, 24, and 60 months. The most commonly reported outcomes included clinical and radiographic success, postoperative pain reduction, pulp vitality preservation, pulp sensibility, hemostasis, quality of life, and long-term treatment outcomes. Overall, the included studies demonstrated considerable clinical and methodological heterogeneity with respect to intervention protocols, biomaterials, outcome measures, and follow-up periods (Table 3).

**Table 3:** Characteristics of Included Studies

Author (Year)	Country	Study Design	Sample size (n)	Population	Intervention	Follow-up	Primary Outcomes
Akhil et al. [13]	India	Randomized controlled trial	60	Mature molars with SIP	Full pulpotomy (MTA)	12 months	Success, pain
Anta et al. [14]	Senegal	Prospective cohort	66	Mature molars with SIP	Full pulpotomy (Biodentine)	12 months	Clinical/radiographic success
Asgary et al. [15]	Iran	Multicenter randomized controlled trial	412	Mature molars with SIP ± apical periodontitis	Full pulpotomy (MTA/CEM)	5 years	Long-term success
Asgary et al. [16]	Iran	Randomized controlled trial	157	Mature molars with SIP	Full pulpotomy	2 years	Success, pain
Baranwal et al. [17]	India	Randomized controlled trial	60	Mature molars with SIP	Full pulpotomy (Biodentine/TheraCal PT)	12 months	Success, pain
Baranwal et al. [18]	India	Randomized controlled trial	50	Mature molars with SIP	Full pulpotomy vs partial radicular pulpotomy	12 months	Success, pulp vitality
Jassal et al. [19]	India	Randomized controlled trial	50	Mature molars with SIP	Partial vs full pulpotomy (Biodentine)	12 months	Success, pain
Jiménez-Martín et al. [20]	Spain	Retrospective cohort	43	Permanent teeth with SIP	Full pulpotomy (calcium silicate material)	24 months	Success, pulp sensibility
Ramani and Sangwan [21]	India	Randomized controlled trial	93	Mature molars with SIP	Complete vs partial pulpotomy	5 years	Long-term success
Shah et al. [22]	India	Randomized controlled trial	60	Permanent teeth with SIP + deep caries	Full vs deep pulpotomy (MTA)	12 months	Success, haemostasis
Suresh et al. [23]	Pakistan	Randomized controlled trial	64	Posterior teeth with SIP	Full pulpotomy (MTA/EBRRM)	6 months	Success, symptom relief
Taha et al. [24]	Jordan	Randomized controlled trial	60	Mature molars with SIP	Full pulpotomy (Biodentine)	12 months	Success, quality of life
Taha et al. [25]	Jordan	Randomized controlled trial	164	Permanent teeth with SIP	Full pulpotomy (MTA/Biodentine/TotalFill)	12 months	Success, pain
Taha and Khazali [26]	Jordan	Randomized controlled trial	50	Mature molars with SIP	Partial pulpotomy (MTA)	2 years	Long-term success
Tzanetakis et al. [27]	Greece	Randomized controlled trial	137	Permanent teeth with SIP	Partial pulpotomy (MTA Angelus/TotalFill BC)	2 years	Success, pain
Zhu et al. [28]	China	Retrospective cohort	81	Adult permanent teeth with SIP	Full pulpotomy (bioceramic material)	12 months	Success, pulp vitality

Risk of Bias Assessment

The methodological quality of the studies included in this review was evaluated using the Cochrane Risk of Bias (RoB 2) tool. Overall, the majority of the randomized controlled trials (RCTs) were considered low risk or with some concerns, primarily because of inadequate randomization, blinding and deviations from intended interventions. Most of the studies had low risk of bias in the outcome measurement and missing outcome data domains, indicating good methodological quality (Table 4).

**Table 4:** Risk of bias assessment of randomized controlled trials using RoB 2

Study	Randomization Process	Deviations from Intended Interventions	Missing Outcome Data	Outcome Measurement	Selection of Reported Results	Overall Risk
Akhil et al. [13]	Some concerns	Low	Low	Low	Low	Some concerns
Asgary et al. [15]	Low	Low	Low	Low	Low	Low
Asgary et al. [16]	Some concerns	Low	Low	Low	Some concerns	Some concerns
Baranwal et al. [17]	Some concerns	Low	Low	Low	Some concerns	Some concerns
Baranwal et al. [18]	Some concerns	Some concerns	Low	Low	Some concerns	Some concerns
Jassal et al. [19]	Low	Low	Low	Low	Low	Low
Ramani and Sangwan [21]	Some concerns	Low	Some concerns	Low	Some concerns	Some concerns
Shah et al. [22]	Low	Low	Low	Low	Low	Low
Suresh et al. [23]	Some concerns	Some concerns	Low	Low	Some concerns	Some concerns
Taha et al. [24]	Low	Low	Low	Low	Low	Low
Taha et al. [25]	Low	Low	Low	Low	Low	Low
Taha and Khazali [26]	Some concerns	Low	Low	Low	Some concerns	Some concerns
Tzanetakis et al. [27]	Low	Low	Low	Low	Low	Low

The Newcastle-Ottawa Scale (NOS) was used to assess the quality of the observational studies. The prospective and retrospective cohort studies included were of satisfactory quality, scoring well in the selection, comparability, and outcome areas. Overall, the observational evidence included in this review was considered methodologically acceptable (Table 5).

**Table 5:** Quality assessment of cohort studies using the Newcastle–Ottawa Scale

Study	Selection (4)	Comparability (2)	Outcome (3)	Total Score (9)	Quality
Anta et al. [14]	4	2	2	8	Good
Jiménez-Martín et al. [20]	4	2	2	8	Good
Zhu et al. [28]	4	2	2	8	Good

Synthesis of findings

Full Pulpotomy Outcomes

Most included studies evaluating full pulpotomy reported high clinical and radiographic success rates in permanent teeth with symptomatic irreversible pulpitis. Favorable outcomes were consistently observed with the use of calcium silicate-based biomaterials, particularly MTA, Biodentine, CEM cement, TotalFill, and bioceramic materials. There were several studies that showed that symptoms were resolved, pulp vitality was maintained, and periapical tissue healed following up periods of 6 months to 60 months. In selected cases, pulpotomy demonstrated outcomes comparable to conventional root canal treatment.

Partial Pulpotomy and Conservative Approaches

Studies evaluating partial pulpotomy, deep pulpotomy, and partial radicular pulpotomy demonstrated outcomes comparable to full pulpotomy in selected cases of irreversible pulpitis. Conservative pulpotomy approaches generally showed favorable success rates, preservation of pulp sensibility, and reduced risk of pulp canal obliteration, supporting their role as minimally invasive alternatives in appropriately selected teeth.

Comparison of Pulpotomy Materials

The clinical and radiographic results of comparative studies with different biomaterials showed similar success rates for MTA, Biodentine, TotalFill, calcium-enriched mixture cement and other bioceramic materials. While most materials gave good results, some studies have reported variations in postoperative pain intensity, hemostasis time and pulp sensibility results, but no significant differences were seen in the degree of success of treatment.

Postoperative Pain and Patient-Related Outcomes

One common finding in all the included studies was the marked reduction in postoperative pain after pulpotomy. Symptom relief was obtained in most patients during the first week following operation. Higher patient satisfaction, better quality of life, shorter treatment duration, and less treatment burden were also observed in the pulpotomy groups compared to the root canal treatment groups in studies comparing pulpotomy and root canal treatment.

Radiographic Outcomes

The majority of studies included showed good radiographic results after pulpotomy, such as no periapical radiolucency or healing of periapical radiolucency, no pathological root changes and no loss of the PDL. A number of studies that used mature permanent teeth with symptomatic irreversible pulpitis showed the radiographic results were consistent for 6 months to 60 months follow-up. Moreover, studies involving teeth with associated apical periodontitis also showed good healing outcomes, reinforcing pulpotomy as a biologically effective treatment and an effective method of maintaining periapical health and promoting tissue healing.

Long-Term Outcomes

Long-term clinical and radiographic studies with up to 60 months follow-up support the long-term success of pulpotomy. Long-term favorable outcomes were observed, including continued pulp vitality, radiographically stable results and resolution of symptoms, confirming that pulpotomy is a long-term predictable treatment option for irreversible pulpitis in permanent teeth.

Discussion

This systematic review synthesized evidence from 16 studies evaluating the clinical and radiographic success of pulpotomy in permanent teeth diagnosed with irreversible pulpitis. Overall, the findings suggest that pulpotomy is a predictable, minimally invasive, and biologically favorable treatment option, demonstrating high success rates across different pulpotomy techniques and biomaterials. The majority of included studies reported favorable outcomes in terms of clinical symptom resolution, radiographic healing, postoperative pain reduction, and preservation of pulp vitality, supporting pulpotomy as a potential alternative to conventional root canal treatment in selected cases.

A major conclusion of this review was the consistently good results with the full pulpotomy for mature permanent teeth with symptomatic irreversible pulpitis. Akhil et al. demonstrated successful outcomes using a cryotherapy-assisted pulpotomy technique, particularly in reducing postoperative pain [13]. Similarly, Anta et al. reported favorable clinical and radiographic success following complete pulpotomy using Biodentine in mature permanent molars at 12 months [14]. Over a 5-year period, Asgary et al. showed long-term clinical and radiographic success without any evidence of recurrence, including in teeth with associated apical periodontitis [15]. Additionally, Asgary et al. showed comparable results to root canal treatment after completion of the pulpotomy, suggesting that conventional root canal treatment may not always be necessary in appropriately selected cases [16].

Additional evidence further strengthened support for conservative pulp therapy approaches. Coronal pulpotomy with Biodentine and TheraCal PT had good outcomes, indicating that modern bioactive materials can assist in achieving good healing and symptom resolution [17]. The similar outcomes obtained from full versus partial radicular pulpotomy further highlighted that attempting to preserve the radicular pulp tissue may not adversely affect the outcomes of treatment and may be beneficial to more conservative management strategies [18]. In addition, comparable clinical and radiographic outcomes between partial and full pulpotomy were reported, suggesting that conservative pulp preservation may be successful when inflammation remains localized and adequate haemostasis is achieved [19]. These findings suggest that conservative pulp preservation can also be successful when the pulp is not extensively involved in the inflammatory process, and good haemostasis is accomplished.

Studies showing favorable long-term results of pulpotomy also supported its long-term predictability. Elective full pulpotomy demonstrated long-term clinical success and maintenance of pulp sensibility in 2-year follow-up periods, in mature permanent teeth with irreversible pulpitis [20]. Likewise, the continued vitality of the pulp, symptom resolution and consistent radiographic findings reported after five years of follow-up confirmed that both complete and partial pulpotomy can be considered definitive treatment options rather than temporary treatments [21]. Additionally, the results of the comparison between deep pulpotomy and full pulpotomy in the extremely deep caries lesions indicated that there is no negative correlation between the amount of pulp tissue preserved and the success rate of the deep pulpotomy treatment, reinforcing the concept of minimally invasive and biologically based approach in endodontic treatment [22].

Another key factor to the success of the treatment was the choice of biomaterial. Better results with either MTA or pre-mixed bioceramic materials indicated that modern calcium silicate-based materials have similar healing potential and clinical predictability [23]. Comparative evidence also revealed that pulpotomy can provide treatment outcomes similar to conventional root canal treatment with added benefits regarding improved quality of life, postoperative pain and patient satisfaction, highlighting the patient-centered benefits of pulpotomy [24]. In addition, there was no significant difference between MTA, Biodentine and TotalFill, which further strengthened the effectiveness of contemporary bioactive materials when it comes to pulp healing and long-term success of the treatment [25].

The success of partial pulpotomy was also supported by Taha et al., who showed good long-term success when MTA was used in cases where the permanent tooth had an irreversible pulpitis diagnosis [26]. Similarly, Tzanetakis et al. reported successful results of partial pulpotomy using MTA Angelus and TotalFill BC with some variation in pain intensity between materials [27]. Zhu et al. also showed that pulpotomy of adult permanent teeth could be achieved with complete clinical and radiographic success after applying a calcium-silicate-based bioceramic material, which is in line with the findings of the others, and confirms that pulpotomy is an effective biologically based treatment option [28].

One of the most consistent findings across the included studies was the significant reduction in postoperative pain following pulpotomy procedures. Some studies indicated that there were significant improvements in symptoms in the first postoperative week, and comparative studies indicated that pulpotomy may result in better immediate postoperative comfort than conventional root canal treatment, with less pain intensity, less treatment time, and higher patient satisfaction. Moreover, the positive radiographic results from the various studies, such as healing of periapical radiolucency, preservation of periapical integrity of the PDL, and lack of further complications, reinforce the clinical predictability of pulpotomy in permanent teeth with irreversible pulpitis.

The results of this review align with the results of previous systematic reviews and recent guidelines for endodontics that recommend vital pulp therapy for irreversible pulpitis in permanent teeth [8,10]. Recent evidence has led to a growing suspicion that irreversible pulpitis does not always require root canal therapy. Rather, the positive results cited in the studies included in this review indicate that pulpotomy could be a viable treatment option to help maintain tooth vitality without the need to remove healthy radicular pulp tissue, consistent with the goals of minimally invasive and biologically oriented dental care.

Several limitations should be noted, however, in spite of the good results. The included studies were highly heterogeneous in study design, pulpotomy technique, biomaterials used, follow-up duration, and outcome criteria, which restricted direct comparisons and prevented quantitative meta-analysis. Further, methodological quality was adequate in general, but some randomized controlled trials showed some concern for risk of bias, especially with regard to the reporting process and randomization.

Further research is recommended, focusing on high-quality multicenter randomized controlled trials with standard protocol, long-term follow-up, and patient-centered outcomes such as quality of life, treatment acceptance, and cost-effectiveness, to further define pulpotomy as a definitive conservative treatment option to RCT in permanent teeth with irreversible pulpitis.

## Conclusions

Pulpotomy appears to be an effective and predictable treatment option for permanent teeth with irreversible pulpitis, demonstrating favorable clinical and radiographic success, including pain reduction, symptom resolution, preservation of pulp vitality, and stable radiographic healing. Full pulpotomy was the most commonly studied technique; however, partial pulpotomy, deep pulpotomy, and partial radicular pulpotomy achieved promising results in selected patients.

Calcium silicate-based materials, particularly MTA, Biodentine, CEM cement, TotalFill, and other bioceramics, demonstrated high success rates and favorable healing potential across studies. Studies also indicate that pulpotomy may have similar results to root canal treatment, but with benefits of reduced postoperative pain, shorter treatment time and increased vitality retention. Additional high-quality multicenter randomized controlled trials with standardized protocols and longer follow-up periods are needed to further establish pulpotomy as a definitive conservative treatment option for irreversible pulpitis.
